# Utility of the Paris System in Urine Cytology for Improved Screening of High-Grade Urothelial Carcinoma in Bahrain

**DOI:** 10.7759/cureus.57189

**Published:** 2024-03-29

**Authors:** Rabbani Mahmoud Daoud, Ali H Ali, Salim Salim Fredericks, Salma Daoud, Hamza R Gomaa, Fatima S AlHashimi

**Affiliations:** 1 Medicine, Royal College of Surgeons in Ireland (RCSI) - Bahrain, Busaiteen, BHR; 2 Emergency Medicine, Salmaniya Medical Complex, Manama, BHR; 3 Biochemistry, Royal College of Surgeons in Ireland (RCSI) - Bahrain, Busaiteen, BHR; 4 General Practice, Albaraka Fertility Hospital, Manama, BHR; 5 Orthopedics and Neurosurgery, Bahrain Defense Force Royal Medical Services, Riffa, BHR; 6 Pathology, King Hamad University Hospital, Busaiteen, BHR

**Keywords:** screening guidelines, high-grade urothelial carcinoma, cellular atypia, urothelial malignancy, the paris system for reporting urinary cytology

## Abstract

Background: Urothelial carcinoma, a prevalent and aggressive urological malignancy, necessitates early detection for improved prognosis. Urine cytology serves as a cost-effective screening tool, but inconsistencies in reporting due to the lack of standardized criteria limit its efficacy. The Paris System for reporting urinary cytology (TPS) was introduced to address this issue, aiming to improve diagnostic accuracy. This retrospective study investigates the effectiveness of urine cytology in detecting high-grade urothelial carcinoma (HGUC) using TPS classification, specifically focusing on atypical urothelial cells (AUC) categorized as TPS-III and suspicious for high-grade urothelial carcinoma (SHGUC) categorized as TPS-IV.

Methods: We reviewed 470 urine cytology samples collected over two years at a tertiary healthcare center in Bahrain. All samples were re-evaluated using TPS classification by two independent consultant cytopathologists blinded to the original cytology report. The analysis included only samples categorized as TPS-III or TPS-IV with corresponding histopathology reports from confirmatory biopsies performed within four months of urine collection. Biopsy results were categorized as either benign/low-grade urothelial carcinoma (non-HGUC) or malignant (HGUC). The positive predictive value (PPV) of urine cytology for HGUC detection was calculated for both TPS-III and TPS-IV categories. Statistical significance was assessed using Fisher's exact test.

Results: Among the 470 urine cytology samples, 40 (8.5%) were classified as TPS-III or TPS-IV. Within this subset, 16 patients underwent confirmatory biopsies. Histopathological analysis revealed HGUC in 12 (75%) patients and non-HGUC (benign or low-grade) in 4 (25%) patients. The PPV of TPS-III for HGUC was 50%, while TPS-IV demonstrated a higher PPV of 90%. However, the difference between these values was not statistically significant (p = 0.25). This study explored the utility of TPS classification in urine cytology for HGUC detection. While SHGUC (TPS-IV) exhibited a numerically higher PPV compared to AUC (TPS-III), the lack of statistical significance necessitates further investigation. Our findings highlight the potential of TPS to improve the accuracy of urine cytology. TPS implementation has been shown to reduce the number of inconclusive "atypical" diagnoses, leading to more targeted investigations.

Conclusion: Our study suggests that SHGUC (TPS-IV) within TPS classification framework might hold promise as a more specific indicator for HGUC compared to AUC (TPS-III). However, further research with larger cohorts is necessary to definitively establish the clinical significance of this observation. This investigation paves the way for future studies exploring the potential of TPS, particularly the SHGUC category, as a reliable screening tool for HGUC, potentially leading to earlier diagnoses and improved patient outcomes.

## Introduction

Urothelial carcinoma is an aggressive urological tumor that primarily affects the bladder, ureters, renal pelvis, and urethra [1]. Globally, it ranks as the fourth most common cancer among males, with an incidence of 82,290 in the United States in 2023 [2] and 573,278 worldwide in 2020 [3]. The most recent cancer registries of the Gulf Cooperation Council (GCC) report that Bahrain had 4758 documented cases of diagnosed urothelial cancer between 1998 and 2012 [4]. In 2022, Al Aradi et al. reported 65 cases of urothelial carcinoma during the period from 2014 to 2018 in the Salmaniya Medical Complex (SMC), the largest tertiary healthcare center in Bahrain, with age-standardized mortality rates (ASR) of 4.59 and 4.58 per 100,000 in males and females, respectively [5].

Urine cytology has emerged as a cost-effective and accessible screening tool for urothelial carcinoma, aimed at facilitating early diagnosis and intervention [6]. However, the lack of universally standardized diagnostic criteria and commonly accepted terminology in urine cytology reporting has resulted in considerable inconsistency among reporting systems. In 2016, pathologists developed a standardized reporting system known as ‘the Paris System for reporting urinary cytology’ (TPS), which divides the urine cytology samples into six categories [7]. The current literature indicates that TPS demonstrates a high sensitivity for high-grade urothelial carcinoma (HGUC) [8]. However, it may have a poor predictive value for diagnosing low-grade urothelial neoplasms [9].

In this retrospective study, we reviewed urine cytology samples collected at a tertiary healthcare center in Bahrain over two years, and reassessed them based on TPS classification, with a specific focus on category III or atypical urothelial cells (AUC) and category IV or suspicious for high-grade urothelial carcinoma (SHGUC). Subsequently, we compared the cytology outcomes as determined by TPS classification to their corresponding biopsy-confirmed histopathology reports as a reference. The primary objective of this study was to assess the diagnostic performance of urine cytology in detecting HGUC, utilizing TPS classification as a standard tool for reporting cytology outcomes, specifically, TPS-III (AUC) and TPS-IV (SHGUC). The aim of this assessment was to potentially pave the way for larger scale cross-sectional studies to investigate the utility of TPS implementation in contributing to early detection and improved screening of urothelial carcinoma. Ultimately, these efforts aim to enhance prognosis and reduce morbidity and mortality rates in the region.

## Materials and methods

Study outline and population

This retrospective cohort study included urine cytology samples obtained from King Hamad University Hospital (KHUH), Bahrain, over a two-year time period from October 2020 to October 2022. A total of 470 samples were included in the study. The inclusion criteria included all patients 18 years or older who presented with symptoms suggestive of urothelial carcinoma (e.g., blood in the urine) and were referred for urine cytology testing as per standard clinical practice at KHUH. Samples with inadequate volume (<10 ml) or improper fixation compromising evaluation were excluded.

Data collection

Data was retrieved from the KHUH electronic medical record system, "HOPE," which stores relevant patient demographics, clinical history, laboratory results including urine cytology reports, and pathology reports. Collected data included patient demographics (age, gender), urine cytology details (specimen number, collection date), biopsy status (documented in HOPE or retrieved from separate pathology reports), and histopathology results, if applicable, specifying benign/low-grade urothelial carcinoma, or LGUC (non-HGUC, or NHGUC) or malignant (HGUC) diagnoses. To ensure objectivity, two independent consultant cytopathologists reviewed the urine cytology samples without access to the original cytology report or any clinical information. Discrepancies in TPS classification (TPS-III, atypical urothelial cells; TPS-IV, suspicious for HGUC) were resolved through joint review and consensus agreement. The KHUH histopathology laboratory database was subsequently queried for all histopathology reports of urine cytology that met the criteria for TPS-III or TPS-IV, from October 2020 to October 2022 to assess the diagnostic performance of the urine cytology findings. Only urine cytology samples categorized as TPS-III or TPS-IV and subsequently confirmed by surgical biopsy with histopathological diagnosis as either benign/LGUC (NHGUC), or malignant (HGUC), within four months of urine collection were included in the final analysis.

Data analysis

The primary outcome measure was the positive predictive value (PPV) of urine cytology for detecting high-grade urothelial carcinoma. Both TPS-III and TPS-IV categories were considered positive for HGUC. PPV was calculated as the ratio of positive cytology results based on TPS classification to the total number of positive results confirmed by biopsy (histopathological diagnosis of HGUC). Fisher's exact test, suited for smaller sample sizes, was employed to compare the ratios of TPS-III and TPS-IV results with confirmed diagnoses. Statistical significance was defined as a p-value ≤ 0.05. Statistical analysis was performed using IBM SPSS Statistics, version 28.0, for Windows (IBM Corp., Armonk, NY).

## Results

Data collected comprised 470 routine urine cytology samples from October 2020 until October 2022. A total of 40 patients (8.5%) meeting the criteria for TPS-III (AUC) and TPS-IV (SHGUC) were identified: 29 (6.17%) were classified as TPS-III and 11 (2.34%) were classified as TPS-IV. In the overall urine cytology group of 470 patients, 55.1% were under 65 years old, 20.2% were in the 65- to 69-year age group, 12.1% were in the 70- to 74-year age group, and 12.6% were aged 75 years and above. The majority were male (72.9%). In the subset of 40 patients with TPS-III or TPS-IV urine cytology reports, the age distribution was more evenly spread with 25.0% under 65 years old, 27.5% between 65 and 69 years of age, 22.5% between 70 and 74 years of age, and 25.0% aged 75 years and above. This group had a higher proportion of males (80.0%). Among the 16 patients in this group who also had histopathology reports, the age distribution was as follows: 18.7% under 65 years, 31.2% between 65 and 69 years of age, 25.0% between 70 and 74 years of age, and 25.0% aged 75 years and above. Notably, all patients in this group were male. The demographic data of the patients are summarized in Table 1.

**Table 1:** Demographic data of the patients showing age and gender distribution

	Original database	Urine cytology group	Urine cytology group plus histopathology
	Frequency (n=470)	Frequency (n=40)	Frequency (n=16)
Age (years)			
Under 65	259 (55.1%)	10 (25.0%)	3 (18.75%)
65-69	95 (20.2%)	11 (27.5%)	5 (31.25%)
70-74	57 (12.1%)	9 (22.5%)	4 (25.0%)
75 and above	59 (12.6%)	10 (25.0%)	4 (25.0%)
Gender			
Male	343 (72.9%)	32 (80.0%)	16 (100.0%)
Female	127 (27.1%)	8 (20.0%)	0 (0.0%)

Our analysis focused on 40 patients classified as either TPS-III or TPS-IV. Among the 40 urine cytology tests performed, only 16 (40%) patients had accompanying follow-up histopathological reports within four months, with 6 being TPS-III and 10 being TPS-IV. Histological diagnosis from biopsies showed 12 (75%) patients with HGUC, and 4 (25%) patients with NHGUC with that being either benign or LGUC. A cyto-histological correlation was performed for the 16 patients to determine the diagnostic performance of the urine cytology samples classified as TPS-III and TPS-IV. The PPV of TPS-III for HGUC was 50% compared to 90% of TPS-IV. A comparison of these ratios (Fisher's exact test) showed no statistical significance between the two groups (p = 0.25) (Table 2).

**Table 2:** Diagnostic performance of AUC (TPS-III) and SHGUC (TPS-IV) TPS: the Paris System for reporting urinary cytology; AUC: atypical urothelial cells; SHUGC: suspicious for high-grade urothelial carcinoma; HGUC: high-grade urothelial carcinoma; NHGUC: non-HGUC; PPV: positive predictive value

TPS diagnostic category	HGUC on biopsy, n (%)	NHGUC on biopsy, n (%)	PPV, %	
AUC (n=6)	3 (50)	3 (50)	50%	
SHGUC (n=10)	9 (90)	1 (10)	90%	

## Discussion

Our study assessed the positive predictive value of urine cytology in detecting HGUC while utilizing the Paris System for reporting urinary cytology as a standardized tool for reporting urine cytology findings, specifically focusing on TPS-III (AUC) and TPS-IV (SHGUC). As we evaluated these two TPS categories as a screening tool for HGUC, it is crucial that they demonstrate a high PPV, indicating increased reliability in accurately identifying individuals with HGUC. The decision to consider AUC as positive cytology for HGUC is based on the current hypotheses regarding the pathogenetic pattern of urothelial carcinoma and TPS criteria description for the said category. The introduction of TPS aimed to restrict the AUC category to cells with atypical features more likely associated with HGUC, which also formed the basis for our interest in establishing its PPV for detecting HGUC. The study findings showed no statistically significant difference in the calculated PPV between the AUC and SHGUC groups. Although the SHGUC group detected a higher proportion (90%) of positive cases compared to the AUC group, definitive conclusions cannot be drawn due to the lack of statistical significance.

Recent studies have reported a frequency of AUC among urine cytology groups to range from 5.76% to 22% [10-13]. However, the term ‘cellular atypia’ has been loosely used by cytologists in the literature to describe urine cytology, with reported frequencies varying between 2% and 31%. This wide variability stems from the non-standardized definition and the lack of structured criteria for ‘cellular atypia’, which often leads to a low inter-observer agreement, over-investigation, and prolonged surveillance. Numerous studies have demonstrated that implementing TPS has reduced the number of cases labeled as AUC [14,15], and increased the PPV for cellular atypia in identifying HGUC. Chan et al. reported a rise in PPV from 43% to 83% after applying TPS criteria [16]. Similarly, Wang et al. demonstrated an increase from 28% to 46% [15]. Bakkar et al. suggested that the adoption of TPS resulted in a 28% decrease in atypical diagnoses within their benign samples, enhancing the clinical significance and accuracy of AUC diagnoses for true cases of HGUC; they also demonstrated an increase in the AUC PPV for HGUC from 43% to 61% following TPS application [13]. While we also anticipated that the rates of atypia would be reduced after the application of TPS in our hospital, unfortunately, the original rate of atypia reports could not be calculated due to missing reports. The frequency of urine cytology cases diagnosed as SHGUC in the current literature ranges from 2.7% to 5.76%, which is lower than our rate at 2.34% [12,17-18]. This discrepancy could be attributed to the larger sample size used in other studies. For instance, de Paula et al. reported a SHGUC frequency of 2.7% among a sample size of 1660 urine specimens [18]. In contrast, our study utilized a smaller sample size of 470, which may account for the lower frequency of SHGUC diagnoses.

The term ‘suspicious‘ has also been widely used by many institutions, including ours, to describe urine cytology, and has always warranted for further investigations and biopsies [19-21]. However, previous reports did not give a clear morphological definition of cells belonging to this category; this is largely due to the lack of a standardized terminology for reporting urine cytology results and the varying criteria applied by pathologists when assigning cases to different categories. As a result, there have been significant interinstitutional variations in the rates of this group reported in various studies [19,22-23]. TPS classification has standardized the morphological definition for SHGUC or TPS-IV, but its PPV for detecting HGUC is still under ongoing assessment.

The existing literature indicates a broad range for the PPV of AUC in detecting HGUC, ranging from 0% to 81%. For SHGUC, the PPV ranges from 35.5% to 100% [13,15-16,24-25]. To the best of our knowledge, our study is the first to specifically focus on the diagnostic performance of SHGUC versus AUC with reference to TPS classification. While our study did not reveal a significant difference between the two categories, it is noteworthy that SHGUC demonstrated a PPV of 90% for HGUC. This is a promising finding that suggests the potential of SHGUC as a reliable indicator for HGUC. However, further studies are warranted to substantiate this possibility and explore the potential of SHGUC as a screening tool for HGUC. These studies should ideally involve a larger sample size to ensure a more comprehensive and robust evaluation of the diagnostic performance of SHGUC.

One of the significant strengths of our study is the use of original cytology slides to assign the Paris System categories, rather than relying on cytology reports. This approach was further strengthened by having two histopathologists evaluate the cytology samples, thereby minimizing the potential for human error. However, our study does have certain limitations. The selection criteria for a four-month follow-up duration between the urine cytology and the biopsy/histopathology report may limit the comprehensiveness of our findings. Other limitations include the relatively small patient sample size and the retrospective nature of data retrieval. To address these limitations and further enhance the validity of our findings, additional studies with larger sample sizes are recommended.

## Conclusions

In conclusion, our study found that the positive predictive value of suspicious for high-grade urothelial carcinoma or TPS-IV for high-grade urothelial carcinoma was 90%, which is higher than the PPV of atypical urothelial cells or TPS-III for HGUC at 50%. However, this difference was not found to be clinically significant. This suggests that while SHGUC may have a higher predictive value for HGUC, the clinical implications of this finding are not yet clear. Therefore, further research is needed to properly evaluate these findings' significance and determine the potential clinical applications of these predictive values.

## References

[1] (2024). What is bladder cancer?. https://www.cancer.org/cancer/bladder-cancer/about/what-is-bladder-cancer.html.

[2] (2024). Cancer stat facts: bladder cancer. Bethesda: National Cancer Institute.

[3] (2024). Bladder cancer: statistics. Alexandria: American Society of Clinical Oncology.

[4] Mubarak M, Jalal A, Ahmadi A, Alaradi H, Altajer A, Awad N (2020). Trends of bladder and prostate cancers. Bahrain Med Bull.

[5] Al Aradi A, Al Rashed AA, Mubarak M, Hasan O, Al Arayedh A, Isa QM, Alaradi H (2022). Renal carcinoma patterns and prevalence in Bahrain: a descriptive study. Cureus.

[6] (2024). Urine cytology: what is it, purpose, procedure & results. Cleveland: Cleveland Clinic.

[7] (2024). Paris system for urothelial neoplasia. Farmington Hills: PathologyOutlines.com, Inc.

[8] Yoo Y, Choi E, Lee DH, Park S, Sung SH (2021). Diagnostic value of urine cytology in detecting histologic variants of urothelial carcinomas in the urinary bladder: cytopathologic correlation of 72 cases. Diagn Cytopathol.

[9] Barkan GA, Wojcik EM, Nayar R, Savic-Prince S, Quek ML, Kurtycz D, Rosenthal DL (2016). The Paris System for reporting urinary cytology: the quest to develop a standardized terminology. Acta Cytol.

[10] Hassan M, Solanki S, Kassouf W, Kanber Y, Caglar D, Auger M, Brimo F (2016). Impact of implementing the Paris System for reporting urine cytology in the performance of urine cytology: a correlative study of 124 cases. Am J Clin Pathol.

[11] Long T, Layfield LJ, Esebua M, Frazier SR, Giorgadze DT, Schmidt RL (2017). Interobserver reproducibility of the Paris System for reporting urinary cytology. Cytojournal.

[12] Dhakhwa R, Shrestha O, Thapa R, Pradhan S (2022). Utility of reporting urine cytology samples as per the Paris system. J Pathol Nep.

[13] Bakkar R, Mirocha J, Fan X, Frishberg DP, de Peralta-Venturina M, Zhai J, Bose S (2019). Impact of the Paris system for reporting urine cytopathology on predictive values of the equivocal diagnostic categories and interobserver agreement. Cytojournal.

[14] Jamwal A, Sharma SK, Sharma N, Raina P, Mahajan A (2020). Correlation of urine cytology for urothelial carcinoma as per Paris system with histopathology. JMSCR.

[15] Wang YJ, Auger M, Kanber Y, Caglar D, Brimo F (2018). Implementing The Paris System for Reporting Urinary Cytology results in a decrease in the rate of the “atypical” category and an increase in its prediction of subsequent high-grade urothelial carcinoma. Cancer Cytopathol.

[16] Chan E, Balassanian R, Tabatabai ZL, Lou H, Vohra P (2018). Improved diagnostic precision of urine cytology by implementation of The Paris System and the use of cell blocks. Cancer Cytopathol.

[17] Yamasaki M, Taoka R, Katakura K (2022). The Paris System for reporting urinary cytology improves the negative predictive value of high-grade urothelial carcinoma. BMC Urol.

[18] de Paula R, Oliveira A, Nunes W (2020). Two-year study on the application of the Paris system for urinary cytology in a cancer centre. Cytopathology.

[19] Muus Ubago J, Mehta V, Wojcik EM, Barkan GA (2013). Evaluation of atypical urine cytology progression to malignancy. Cancer Cytopathol.

[20] Serretta V, Pomara G, Rizzo I, Esposito E (2000). Urinary BTA-Stat, BTA-Trak and NMP22 in surveillance after TUR of recurrent superficial transitional cell carcinoma of the bladder. Eur Urol.

[21] Trott PA, Edwards L (1973). Comparison of bladder washings and urine cytology in the diagnosis of bladder cancer. J Urol.

[22] Brimo F, Vollmer RT, Case B, Aprikian A, Kassouf W, Auger M (2009). Accuracy of urine cytology and the significance of an atypical category. Am J Clin Pathol.

[23] Nabi G, Greene DR, O’Donnell M (2003). How important is urinary cytology in the diagnosis of urological malignancies?. Eur Urol.

[24] Snow JT, McIntire PJ, Siddiqui MT (2018). The Paris System: achievement of a standardized diagnostic reporting system for urine cytology. Diagn Histopathol.

[25] Nguyen L, Nilforoushan N, Krane JF, Bose S, Bakkar R (2021). Should "suspicious for high-grade urothelial carcinoma" and "positive for high-grade urothelial carcinoma" remain separate categories?. Cancer Cytopathol.

